# Improving triplet lamb survival: management practices used by commercial farmers

**DOI:** 10.3389/fvets.2024.1394484

**Published:** 2024-07-30

**Authors:** Cathrine Erichsen, Tamsin Coombs, Neil Sargison, Sue McCoard, Tim W. J. Keady, Cathy M. Dwyer

**Affiliations:** ^1^Department of Animal and Veterinary Sciences, Scotland's Rural College (SRUC), Edinburgh, United Kingdom; ^2^The Royal (Dick) School of Veterinary Studies, University of Edinburgh, Edinburgh, United Kingdom; ^3^AgResearch Ltd, Grasslands Research Centre, Palmerston North, New Zealand; ^4^Teagasc, Animal and Grassland Research and Innovation Centre, Athenry, Ireland

**Keywords:** sheep, lamb survival, triplet lambs, management, survival, farmers, attitudes

## Abstract

**Introduction:**

Prolificacy has become an important breeding goal in sheep farming to increase farm profitability. With the adoption of improved genetics and management practices leading to increased lambing percentages, the proportion of triplet-born lambs has also increased on farms. However, mortality rates of triplet lambs are higher than for single- and twin-born lambs, and additional management inputs may be needed to support survival. The aim of this study was to identify factors that affect management practices that are considered important for triplet lamb survival by commercial farmers from the United Kingdom (UK), the Ireland (IRE), and New Zealand (NZ).

**Methods:**

An online survey was developed and disseminated to farmers in each country, focusing on farmer demographics, flock characteristics, management practices and production outcomes. A total of 448 farmers completed the survey, from the UK (*n* = 168), IRE (*n* = 218), and NZ (*n* = 62).

**Results:**

Respondents had larger flocks, higher scanning and lambing percentages than the country average for the UK and IRE. The mean percentage of triplet litters born within flocks was 9%, and lambs lost between scanning and lambing were 14% for UK, 15% for IRE, and 25% for NZ respondents (*P* = 0.063). Overall, 60% of all respondents reported to lamb indoors and 40% lambed outdoors, however NZ farmers almost exclusively lambed outdoors, whereas UK and IRE farmers lambed in both systems (*P* < 0.001). NZ farmers were more likely to rear all triplet lambs with the ewe, whereas UK and IRE farmers were more likely to remove a lamb to rear by another ewe or artificially (*P* < 0.001). Factors that influenced triplet lamb management practices of respondents in this study were respondent country of origin, flock size, age, and gender. In general, younger respondents (*P* < 0.001), and female respondents (*P* < 0.05), were more likely to engage in management activities that were considered to promote better triplet lamb survival, compared to older and male respondents respectively. These practices were associated with better lamb survival reported by respondents but were less likely to be carried out when flock size increased (*P* < 0.001).

**Discussion:**

The results of this survey highlight future priorities or communication strategies needed to improve triplet lamb survival.

## 1 Introduction

Prolificacy is the main factor influencing the profitability of prime lamb production and has also become an important breeding goal in sheep farming due to a growing worldwide demand for animal products (1, 2). This has resulted in more lambs born per ewe, and a greater proportion of ewes giving birth to triplet lambs (3, 4). However, lamb mortality rates are significantly higher in triplet-born lambs compared to singles and twins (5, 6). This is a welfare issue and important cause of wastage that affects efficiency, profitability and eco-efficiency of sheep farming (7–10).

Multiple factors influence survival of triplet-born lambs (11). In comparison to twin-born lambs, triplet-born lambs have lower birthweights, are at a higher risk of failing to thermoregulate and are slower to find their dam's udder and suckle (6, 12–15). These characteristics decrease chances of survival in the first 7 days *postpartum* (16). Furthermore, triplet-bearing ewes experience greater risks of metabolic upset such as pregnancy toxemia (17), and may also have a greater risk of experiencing dystocia than twin-bearing ewes (18, 19). Thus, the risks to health and welfare experienced by triplet-bearing ewes and their lambs may require tailored management systems during pregnancy and at lambing to improve ewe and lamb survival, productivity, and profitability.

Sheep farming systems worldwide have adopted different management styles and practices at lambing depending on their location and available resources. This has resulted in very distinct ways of sheep farming. For example, ewes may be managed indoors or outdoors during lambing and strategic feeding management systems may be implemented in mid-late gestation based on expected litter size and ewe body condition (20). In areas where grass is scarce during winter, or perceived risks from predation is high, many sheep flocks are kept indoors from approximately mid-pregnancy until lambing or for part of lactation. In these higher-input systems, there are more opportunities to provide tailored nutritional management of pregnant ewes and to intervene with animals that have difficult births or provide interventions which protect the newborn lamb from hypothermia and starvation (21). However, indoor systems may limit natural behaviors such as seeking a birth site, require greater resource inputs (e.g., infrastructure, labor, feed, and energy) and have a greater risk of animal health issues than outdoor systems (22, 23). Low input lambing systems have been developed to enable ewes to lamb and rear at least one lamb with a minimum amount of human interference (24). In the early 1960's, New Zealand (NZ) sheep farmers developed an “easy-care” system where ewes and rams were selected for lambing ease and the ability to rear their lambs without assistance (25). The temperate climate in NZ provides an opportunity for farmers to keep ewes on pasture throughout the year and ewes can be set stocked and largely left alone for lambing. The greatest risks of lamb mortality in NZ and outdoor lambing systems worldwide are starvation, mismothering and hypothermia (20).

Research in recent years has focused on triplet lamb survival and physiological differences in triplet-born lambs compared to twins (11). However, whether or not farmers with high lambing percentages, defined as number of lambs per number of ewes put to the ram, adjust their management and labor input to meet the specific needs of ewes and their triplet lambs, has not been investigated so far. Dwyer et al. highlighted that lamb survival rates have not changed despite the increase in scientific research on the topic (5), which may reflect low levels of uptake of research findings on sheep farms.

The United Kingdom (UK), Ireland (IRE), and NZ have a great impact on global trade in sheep meat (26). Thus, increased prolificacy may be an important breeding goal in these countries to produce more lambs for slaughter. These countries also represent predominantly pasture-based prime lamb production but with very distinct lambing systems, which could reflect differences in triplet lamb management. This work grew from an EU Horizon 2020 funded thematic network, SheepNet^1^ (Sharing Expertise and Experience toward sheep Productivity through NETworking), which aimed to share expertise between sheep farmers in different countries and regions. Outcomes from that project identified that farmers liked to learn from other farmers, and thus sharing practices across countries can be valuable methods to improve management, and ewe productivity. The aim of this study was, therefore, to investigate different management practices that could have value and improve triplet survival when shared in different systems. Surveys can be used to reach a broad spectrum of farmers to help identify both management practices and drivers to adopt new practices in sheep farming. Through survey analysis, it is possible to collect large amounts of data from a potentially broad audience of farmers. This method has previously been conducted to identify management practices related to general lamb survival (27, 28) and drivers to adopt new technologies in sheep farming (29). In this study, an online questionnaire was disseminated to farmers from the UK, IRE, and NZ with the objective to identify factors that affect on-farm management practices during pregnancy and at lambing that are considered important for triplet lamb survival. We specifically focused on pregnancy management and the first 3 days after lambing as this is known to be the greatest period of risk for lamb mortality (5).

## 2 Methods

A questionnaire was designed and administered exclusively through the web-based questionnaire system “Jisc Online Surveys” (Jisc, 2020^2^, https://www.onlinesurveys.ac.uk/) and disseminated to UK, IRE, and NZ farmers with sheep from November 2019 until March 2020. Prior to distribution, the questionnaire was piloted by 6 UK, IRE, and NZ farm consultants, farmers, and scientists to identify possible issues of wording and understanding. Thereafter, wording was amended to enable understanding in all countries. The questionnaire was approved by the Royal (Dick) School of Veterinary Studies Human Ethical Review Committee (approval number: HERC_415_19), and AgResearch Human Research and Ethical Conduct (approval number: 14/2019). The questionnaire was promoted through newspapers and media releases from SRUC, the Royal (Dick) School of Veterinary Studies, Teagasc and AgResearch, national and international Facebook pages for sheep farmers, industry networks and websites. It started with a brief outline of the research aim and consent. Thereafter, 34 questions were divided into three sections. To help identify factors that could influence lamb survival, the first section asked sociodemographic questions which focused on respondents' age, gender, and number of years' experience in sheep farming and flock sizes. Respondents were then asked about production data such as scanning percentages (defined as number of lambs identified as present as fetuses when the ewes were scanned by ultrasonography in mid pregnancy per number of ewes put to the ram × 100), lambing percentages (defined as number of lambs (born or present at 12 weeks) per number of ewes put to the ram × 100), and number of sets of triplets or greater born on farm, to be able to investigate direct outcomes of on-farm practice. The second section focused on management practices of triplet-bearing ewes during pregnancy and ewes and triplet lambs in the first 3 days after birth. The third part of the questionnaire focused on attitudes of farmers to profitability of rearing triplet lambs, the resources needed, triplet survival and the welfare of the ewe and triplet lambs (Q30–34). The results from the third section will be published in a subsequent publication.

The questions were predominantly closed-ended and measured on a 5-point Likert scale (“I strongly disagree,” “I disagree,” “Neither agree nor disagree,” “I agree,” “I strongly agree”). A 5-point Likert scale was chosen to allow respondents to provide a neutral response. Some open-ended questions were also asked to allow farmers to expand on their answers, for example, to add a breeding aim that was not specified on the list, or to expand on why they had answered in a certain way in a previous question. These responses were then re-categorized for further analysis where possible (for example by forming new categories or assigning to existing categories). The questions all related to the farmers' recollection of their last lambing season, which for all countries would have been in 2019. A copy of the complete survey is shown in the Supplementary material.

### 2.1 Statistical analysis

In total 510 respondents took part in the questionnaire. The responses of farmers with <50 breeding ewes (*n* = 62) were considered to represent “lifestyle” farming or those where lamb production was not an important commercial aim of the business and thus may be less likely to engage in triplet lamb management practices of broader relevance to commercial farmers. These responses were excluded from analysis leaving 448 respondents in the dataset with commercial-scale sheep production.

The data were entered into MS Excel (Microsoft, Redmont, WA, USA). Some categories were regrouped prior to analysis (see Supplementary material for details). The mean percentage of lambs lost between scanning and lambing was derived from figures reported by each individual respondent.

*P*-values of ≤ 0.05 were considered statistically significant. *Post-hoc* Tukey tests were conducted to determine differences between the three countries. The results are presented as means and ± standard error of the mean (SE). If data had been transformed the back-transformed mean and upper and lower 95% confidence interval (CI) are reported. Respondent information (gender, age, experience, and education) and farm information (farm type, type of flock, lambing system, and flock size) were examined with descriptive statistics (percentages and frequencies) and analyzed with Kruskal-Wallis tests. Binary Logistic Regression was conducted to analyze the relationship between country (“UK,” “IRE,” or “NZ”), gender (“male” or “female” respondents), age (“ ≤ 44 years” or “≥45 years”), experience (“ ≤ 10 years” or “≥11 years”), flock size, and lambing system (“indoor” or “outdoor”). As the main predictor of lambing system was country, within country differences were analyzed (Table 1). For UK and IRE respondents, analyses of indoor and outdoor systems were conducted while only data from NZ respondents with outdoor lambing systems were analyzed, as too few NZ respondents (3/62, 5%) provided information on indoor lambing.

**Table 1:** Results of Binary Logistic Regression to investigate predictors of lambing system (indoor vs. outdoor housing).

**Predictor**	**A**	**B**	**S.E**.	**Wald**	**df**	***P***	**Odds ratio**	**95% CI**
Country	NZ	IRE		37.29	2	<0.01		
							0.033	0.008; 0.136
	UK	IRE					0.692	0.424; 1.1308
	UK	NZ					20.936	5.465; 80.201
Gender	Female	Male	0.267	1.14	1	0.287	1.331	0.789; 2.244
Age	≤ 44 years	≥45 years	0.240	1.36	1	0.244	1.322	0.827; 2.115
Experience	≤ 10 years	≥11 years	0.259	2.13	1	0.144	1.461	0.880; 2.427
Flock size			0.000	111.67	6	0.243	0.998	0.999; 1.000

Separate questions in the questionnaire covered the topics of pregnancy and triplet lamb management. Descriptive analysis of these data were undertaken by country and by lambing practice (indoor or outdoor). To avoid Type I errors, these responses were then combined into composite “Best Practice” scores for more detailed analysis. Scores were developed based on internationally peer-reviewed literature to identify which factors influence management practices. The main aim of these “Best Practice” scores was to focus on the best outcome for triplet-born lambs to be born alive, with adequate energy reserves, free from birth stress, able to intake colostrum, and to receive assistance when needed. Furthermore, the “Best Practice” scores were applied to address the specific needs triplet-bearing ewes and triplet lambs may have compared to single- and twin-born lambs. The scores developed were “Best Pregnancy Practice” (Table 2), “Best Triplet Lamb Practice” (Table 3), and overall “Total Best Practice” which summed pregnancy and triplet lamb management. The scores were developed as an additive model, meaning that each criterion was counted equally as 1 if respondents had reply “yes,” and 0 when they had replied “no.” The maximum score for “Best Pregnancy Practice” was 7, for “Best Triplet Lamb Practice” was 6, and for “Total Best Practice” was 13. It is recognized that not all farmers fulfilled all scores for the Best Practice scores.

**Table 2:** Topics important for pregnancy management of triplet-bearing ewes to create the Best Pregnancy Practice score.

**Questions of interest for Best Pregnancy Practice**	**References**	**Score^a^**
If respondents are pregnancy scanning, they scan for litter size or Litter size and fetal age	(30)	1
The respondent is body condition scoring by manual palpation	(31)	1
Ewes are fed according to litter size	(11)	1
Ewes are managed according to body condition score (BCS)	(32)	1
Triplet-bearing ewes are kept in separate groups from twins and singles	(33)	1
	(34)	
Ewes are frequently inspected	(11)	1
Ewes are supplemented in the last 4–6 weeks of pregnancy	(33)	1
	(35)	
**Total**	7	

^a^Score refers to the points awarded if the respondent answered “yes” to the question, these were additive for the Best Pregnancy Practice score.

**Table 3:** Topics important for triplet lamb management and management of triplet-bearing ewes to create the Best Triplet Lamb Practice score.

**Questions of interest for Best Triplet Lamb Practice**	**References**	**Score^a^**
Triplet lambs are moved to an individual pen after birth	(22)	1
Ewes with their triplet lambs are provided with shelter	(36)	1
Triplet lambs are assisted when they have not sucked	(37)	1
Lambs are assisted by holding them to the udder	(38)	1
The preferred supplement is ewe colostrum	(39)	1
Lambs and ewes are inspected hourly or several times a day	(22)	1
	(37)	
**Total**	6	

^a^Score refers to the points awarded if the respondent answered “yes” to the question, these were additive for the Best Pregnancy Practice score.

The Best Practice Scores were analyzed in Genstat (Genstat for Windows version 19th Edition. VSN International, Hemel Hempstead, UK). Prior to analysis the data were deemed suitable without transformation or log10 transformations were performed (% triplet lambs in the flock) and a linear mixed model [residual maximum likelihood analysis (REML)] was used on all data. REML analysis does not require balanced sample sizes and was therefore suitable for this study. Initial univariate analyses of the Best Practice Scores were conducted to identify the fixed effects and covariates for the final analyses. The criteria to be fitted in the final model was *P* ≤ 0.2. Country, gender, age and experience were analyzed as fixed effects, flock size as covariate and URN (Uniform Resource Number, identity code for each respondent) as a random effect. Breed of sheep was not included in the analysis. The model was then re-run to robustly test the effect of the remaining terms in the model. The Best Practice Scores for the respective countries were suitable for REML analysis in the same manner as the analyses of country comparisons.

Outcome measures related to survival [scanning percentage, lambing percentage, percentages of triplets born, percentage of lambs lost (lamb mortality) were analyzed using REML with univariate analyses to identify the final models, but with the Best Practice Scores and flock size added as covariates. Lambing percentage was also included as a covariate for the analysis of lamb mortality]. The production statistics (scanning and lambing percentage, number of triplet litters and lambs lost between scanning and lambing) for the individual countries were not normally distributed and could not be transformed and were therefore analyzed with a generalized linear mixed model (GLMM). The same fixed effects and coefficients were used as previously.

Responses to questions about satisfaction with their triplet lamb management and whether or not triplet lambs were wanted were analyzed separately. Neither variables were suitable for REML analysis as the data were skewed and could not be normalized. Hence, a GLMM was conducted with Poisson distribution and logarithm function. The effect of respondent age, gender and experience on the satisfaction with their triplet lamb management included flock size, Best Pregnancy Practice, Best triplet Lamb Practice and Total Best Practice as covariates. For the response variable whether triplet lambs were wanted by the respondents, lambing percentage, percentage of triplets, Total Best Practice were included as covariates and age of respondent as fixed effect.

## 3 Results

### 3.1 Demographics

The overall demographic data for respondents is presented in Table 4. Of the 448 respondents, 38% (168) came from the UK, 49% (218) from IRE and 14% (62) from NZ. Three-quarters of respondents to the survey were male, while IRE had fewer female respondents compared to UK and NZ (*P* < 0.001). Overall, respondents were approximately equally split between those who were younger than 44 years and those who were older than 45 years of age. More than 50% of UK and NZ respondents were 44 years and younger, whereas for IRE more than 60% of respondents were 45 years and older (*P* < 0.001). Furthermore, 79% of respondents reported undertaking some additional formal education after leaving school (further education and university level degrees). Overall, 70% of respondents had more than 10 years of experience of working with sheep.

**Table 4:** Overall and country demographics for participating respondents.

		**UK**	**IRE**	**NZ**	**Total**	**Significance**
Gender	Females	40% (67)^a^	12% (25)^b^	28% (17)^a^	24% (109)	Kruskal-Wallis = 41.77, *P* < 0.001, df = 2
	Males	60% (101)^a^	89% (192)^b^	72% (44)^a^	76% (337)	
Age of respondent	≤ 44 years	56% (94)^a^	39% (86)^b^	61% (38)^a^	49% (218)	Kruskal-Wallis = 16.53, *P* < 0.001, df = 2
	≥45 years	44% (74)^a^	61% (132)^b^	39% (24)^a^	51% (230)	
Experience	≤ 10 years	29% (49)	27% (59)	40% (25)	30% (133)	Kruskal-Wallis = 4.09, *P* = 0.129, df = 2
	≥11 years	71% (119)	73% (150)	60% (37)	70% (315)	
Education	High school	20% (34)	17% (37)	29% (18)	20% (89)	Kruskal-Wallis = 0.31, *P* = 0.855, df = 2
	Further education	31% (52)	39% (86)	21% (13)	34% (151)	
	University level	48% (80)	41% (90)	50% (31)	45% (201)	
	Other	1% (2)	2% (5)	0	2% (7)	

Data are presented as percentages and number of respondents within country.

Superscripts with different letters indicate significant (P ≤ 0.05) differences.

Of the respondents, a third farmed (raised) only sheep, whereas 48% farmed sheep and beef cattle, and 18% farmed sheep and something else such as cropping or deer (Table 5). Most (73%) of respondents had commercial sheep flocks, or flocks that were a mix of commercial and pedigree (breeding) animals (22%). Relatively few respondents (5%) were stud or pedigree breeders. Respondents farmed with a wide range of different sheep breeds: UK farmers used 42 different breeds (and their crosses), IRE farmers used 26 breeds and NZ farmers 22. Composite breeds and commercial crossbreds were also used by farmers in all locations although the specific breeds or types present in these animals were not always specified. Across countries common breeds were Romney, Dorset, Suffolk, Texel, and Cheviot, which were used by farmers in all countries, but some breeds were unique to the different locations. In IRE Belclare, Charollais, and Lleyn were also commonly mentioned, and in UK Lleyn and Scottish Blackface were also frequently cited. Hill, upland and lowland type breeds were represented in all countries, suggesting that respondents farmed a variety of different systems. Although some farmers reported only one breed, most used several breeds and crosses and up to seven different breeds were used on some farms.

**Table 5:** Farm information for respondents^*^.

		**UK**	**IRE**	**NZ**	**Total**	**Significance**
Farm type	Sheep	35% (58)	36% (79)	24% (15)	34% (152)	Kruskal-Wallis = 3.20, *P* = 0.202, df = 2
	Sheep and beef	41% (68)	50% (108)	61% (38)	48% (214)	
	Sheep and something else	24% (42)	14% (31)	15% (9)	18% (82)	
Type of flock	Commercial	60% (100)^a^	84% (182)^b^	73% (45)^ab^	73% (327)	Kruskal-Wallis = 16.22, *P* < 0.001, df = 2
	Stud/pedigree	8% (13)^a^	3% (6)^b^	8% (5)^ab^	5% (24)	
	Commercial and stud/pedigree	33% (55)^a^	14% (30)^b^	19% (12)^ab^	22% (97)	
Lambing system	Indoor	61% (103)^a^	74% (161)^b^	5% (3)^c^	60% (267)	Kruskal-Wallis = 95.60 *P* < 0.001, df = 2
	Outdoor	39% (65)^a^	26% (57)^a^	95% (59)^b^	40% (181)	
Flock size		539 ± 49^a^	211 ± 13^b^	2,640 ± 270^c^	670 ± 57	Kruskal-Wallis = 138.3, *P* < 0.001, df = 2

^*^Superscripts with different letters indicate significant (P ≤ 0.05) differences.

Overall, 60% (267) respondents reported lambing indoors, while 181 (40%) respondents lambed outdoors. IRE respondents had the highest proportion of indoor lambing flocks (74%), and NZ the lowest (5%) with UK intermediate (61%; Table 5; *P* < 0.001). The overall average flock size was 670 ± 56.6 breeding ewes. However, NZ respondents had a mean flock size that was nearly five times that of UK farmers, and the UK respondents had flocks that were more than twice as large as IRE respondents (Table 5; *P* < 0.001).

**Figure 1:**
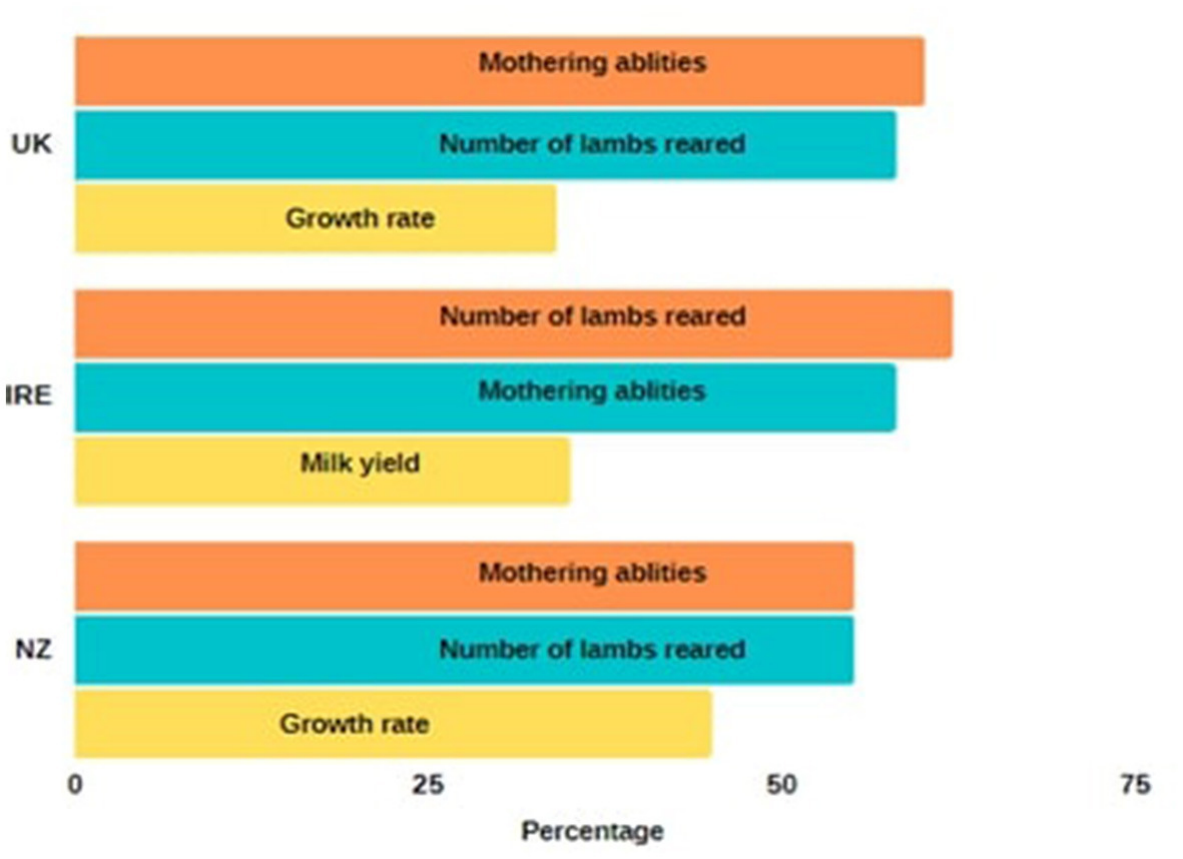
Top 3 (orange = first priority, blue = second priority, and yellow = third priority) breeding goals for respondents from United Kingdom (UK), Republic of Ireland (IRE), and New Zealand (NZ). Data are presented as percentages who chose the particular attributes.

Of the 14 options presented, respondents from the three countries had very similar breeding goals with each country choosing mothering abilities and number of lambs reared as part of their top three breeding goals. Growth rate was also selected in the UK and NZ whereas IRE respondents chose ewe milk ability as their third priority of breeding goals (Figure 1), although these attributes are clearly linked.

**Figure 2:**
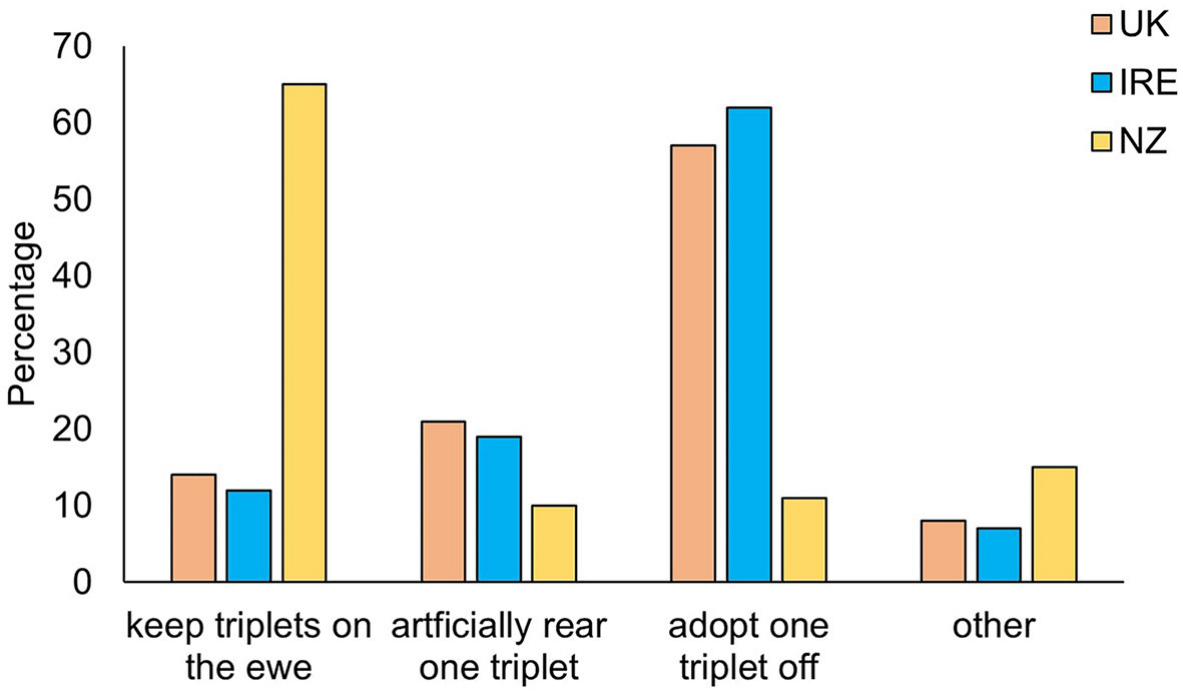
Management practices for triplet-born lambs for respondents from United Kingdom (UK), Republic of Ireland (IRE), and New Zealand (NZ; Kruskal-Wallis = 38.85, *P* < 0.001, df = 2). Data are presented as percentages of respondents who selected the different practices.

When asked about their triplet rearing practices, respondents from NZ (65%) were more likely to keep all triplet lambs on the ewe compared to UK (12%) and IRE (14%; Figure 2, *P* < 0.001). Respondents from the UK (57%) and IRE (62%) were more likely to adopt a lamb onto another ewe or artificially rear one triplet (Figure 2, P <0.001). When asked which lamb would typically be adopted from the litter more than half of the respondents said the smallest/lightest (54%), although a third of respondents (32%) said they would choose a medium sized lamb. Smaller numbers of respondents chose the heaviest lamb (8%) or the weakest lamb (5%). No respondents selected the most vigorous lamb or made their choice based on the lamb's sex. There was however a significant difference by country (X^2^ = 59.03, d.f. = 6, *P* < 0.001): NZ respondents were more likely to remove a medium sized lamb (54.2%) than UK or IRE respondents (35.8 and 23.9%, respectively), and were also more likely to select the weakest lamb than UK or IRE respondents (NZ = 16.9%, UK = 8.5%, IRE = 0.5%). The most frequently expressed choice of a triplet lamb to remove for both UK and IRE was the smallest or lightest lamb (UK = 48.5%, IRE = 66.8%).

### 3.2 Production statistics

Overall, independent of country, scanning and lambing percentages were 182 ± 1.2 and 166 ± 1.2, respectively. There was no significant difference between countries in reported scanning percentage, but UK respondents tended to report a higher lambing percentage than other countries (*P* = 0.08, Table 6). The mean percentage of all lambs lost, independent of litter size, between scanning and lambing (derived from figures reported by respondents) was 15% ± 0.9. However, NZ respondents reported higher lamb mortality rates from scanning to 12 weeks of age (25%) compared to respondents from the UK (14%) and IRE (15%) respondents (*P* = 0.063, Table 6). The reported mean percentage of triplet litters born in the flock in the last lambing period was 9% ± 0.04. NZ and IRE respondents reported significantly more sets of triplets (7 and 9%, respectively, compared to UK (11%, *P* = 0.037, Table 6).

**Table 6:** Results of production data reported as mean and standard error, or confidence interval (CI) for UK, IRE, and NZ respondents^*^.

	**UK**	**IRE**	**NZ**	**Total**	**Significance**
Scanning percentage	184 ± 2.0	183 ± 1.8	176 ± 4.7	182 ± 1.2	*F*_2, 398_ = 0.94, *P* = 0.390
Lambing percentage	168 ± 3.8	162 ± 3.9	162 ± 5.5	166 ± 1.2	*F*_2, 426_ = 2.52, *P* = 0.080
Lambs lost between scanning and 12 weeks (%)	14 ± 1.3	15 ± 1.1	25 ± 3.5	16 ± 0.9	*F*_2, 354_ = 2.78, *P* = 0.063
Triplet-litters (% of flock)	7 [6.6;7.6]^a^	9 [7.9;9.2]^b^	11 [9.3;12.2]^b^	9 ± 0.04	*F*_1, 415_ = 3.34, *P* = 0.037

^*^Superscripts with different letters indicate significant (P ≤ 0.05) differences.

Overall, male respondents reported higher lambing percentages (Male: 167 ± 3.7, Female: 161 ± 4.1, *P* = 0.032) and more triplets [Male: 10% [9.2; 10.4], Female: 7% [6.8; 8.2], *P* = 0.010] than female respondents. There were no significant effects of respondent gender on the production statistics investigated within countries.

There were no overall effects of respondent age on the reported production statistics. The only respondent age effect within countries was found for IRE indoor systems where older IRE respondents reported higher lamb mortality rates than younger respondents [≥45 years: 19% [16.5; 21.3], ≤ 44 years: 14% [12.4; 15.9], Wald = 4.43, df = 1, *P* = 0.035].

Overall, an increase in flock size was associated with a decreased lambing percentage (β = −0.0038, *P* = 0.006). In UK outdoor systems, increasing flock size was associated with a lower lambing percentage (β = −0.0001, *P* = 0.017) and a decreased percentage of triplets in flocks (β = −0.0006, *P* < 0.001). An increase in flock size was also associated with increased lambs lost between scanning and 12 weeks of age (β = 0.0024, *P* = 0.027) and similar results were found for IRE- (β = 0.0007, *P* = 0.015) and UK indoor systems (β = 0.0003, *P* = 0.010).

### 3.3 Management practices and best practice scores

The types of management practices undertaken by farmers in pregnancy (Table 7) and at lambing time (Table 8) are shown for the different countries (Tables 7A, 8A) and by indoor and outdoor lambing (Tables 7B, 8B). Overall, for pregnancy management, the majority of farmers scanned their ewes for litter size, carried out measurements of BCS using manual palpation, used BCS to manage the ewes, fed their ewes for litter size, and inspected the ewes more than once a day. There were few differences between management of pregnant ewes between respondents who lambed indoors or outdoors except those who lambed indoors were more likely to feed the ewes for litter size than those who lambed outdoors (X^2^ = 3.95, d.f. = 2, *P* < 0.05). There were few significant differences between farmers from different countries in specific aspects of their pregnancy management, although UK respondents were more likely to BCS manually than IRE respondents (X^2^ = 28.25, d.f. = 2, *P* < 0.001) and both UK and IRE respondents were more likely to pregnancy scan for litter size than NZ respondents (X^2^ = 38.30, d.f. = 3, *P* < 0.001). However, NZ farmers differed markedly from UK and IRE respondents in whether they sheared ewes prior to lambing: nearly half of NZ respondents sheared before lambing, whereas only 9 and 3.5% of IRE and UK respondents did so, respectively (Table 7A: X^2^ = 82.28, d.f. = 2, *P* < 0.001).

**Table 7:** Impact of (a) country and (b) lambing management on different pregnancy management practices.

**Practice**	**IRE**	**UK**	**NZ**	**Overall**
**(A)**				
BCS manually	89 (40.83)	109 (64.88)	32 (51.6)	230 (51.34)
Preg scan litter size	143 (71.50)	119 (78.81)	25 (45.45)	287 (70.69)
Fed litter size	175 (80.28)	131 (77.98)	42 (67.74)	348 (77.68)
Manage by BCS	109 (50.00)	97 (57.73)	39 (62.90)	245 (54.69)
Triplets separate	146 (66.97)	114 (67.86)	34 (54.84)	294 (65.63)
Frequent inspection^a^	94 (43.11)	86 (51.19)	36 (58.06)	216 (48.21)
Shorn prior to lambing	20 (9.17)	6 (3.57)	29 (46.77)	55 (12.28)
**(B)**				
**Practice**	**Lambing indoors**	**Lambing outdoors**	***P***	
BCS manually	135 (50.56)	95 (52.49)	NS	
Preg scan litter size	180 (72.87)	107 (67.30)	X^2^ = 3.54, d.f. = 3, NS	
Fed litter size	216 (80.90)	132 (72.93)	X^2^ = 3.95, d.f. = 2, *P* < 0.05	
Manage by BCS	148 (55.43)	97 (53.59)	NS	
Triplets separate	183 (68.54)	111 (61.33)	NS	
Frequent inspection^a^	127 (47.57)	89 (49.17)	NS	
Shorn prior to lambing	23 (8.61)	32 (17.68)	X^2^ = 8.23, d.f. = 2, *P* = 0.005	

In each case, where there were multiple responses, the practice considered most important for good management in each case is presented.

^a^More than once per day.

**Table 8:** Impact of (a) country and (b) lambing management on different triplet lamb management practices, in each case, where there were multiple responses, the practice considered most important for good management in each case is presented.

**Practice**	**IRE**	**UK**	**NZ**	**Overall**
**(A)**				
Left alone at lambing (all)	33 (15.14)	35 (20.83)	22 (35.48)	90 (20.09)
Left alone at lambing (singles)	18 (8.25)	8 (12.90)	15 (8.93)	41 (9.15)
Left alone at lambing (none)	164 (75.23)	118 (70.24)	29 (46.77)	311 (69.42)
Always allowed to lamb at birth site^a^	120 (55.05)	105 (62.87)	47 (75.81)	272 (60.85)
Individual pen after lambing (all)	**146 (67.28)**	**101 (60.84)**	**2 (3.23)**	249 (55.96)
Individual pen after lambing (triplets)^b^	36 (16.59)	31 (18.67)	3 (4.84)	70 (15.73)
Individual pen after lambing (none)	**27 (12.44)**	**31 (18.67)**	**57 (91.94)**	115 (25.84)
Shelter provision (all)	123 (56.42)	117 (69.64)	22 (35.48)	262 (58.48)
Routine colostrum supplement (all)	**168 (77.06)**	**132 (78.57)**	**14 (22.58)**	314 (70.09)
Routine colostrum supplement (triplets)^b^	14 (6.42)	19 (11.31)	4 (6.45)	35 (7.81)
Routine colostrum supplement (none)	**28 (12.84)**	**11 (6.55)**	**44 (70.97)**	83 (18.53)
Inspection at lambing several times a day	**213 (97.71)**	**158 (94.05)**	**15 (24.19)**	386 (86.16)
Inspection once a day or less at lambing	**0 (0)**	**1 (0.60)**	**30 (48.39)**	31 (56.92)
Lambs assisted to suck if required	**216 (99.08)**	**167 (99.40)**	**36 (58.06)**	419 (93.53)
**(B)**				
**Practice**	**Lambing indoors**	**Lambing outdoors**	***P***	
Left alone at lambing (all)	34 (12.73)	56 (30.94)		
Left alone at lambing (singles)	22 (8.24)	19 (10.50)		
Left alone at lambing (none)	210 (78.65)	101 (55.80)		
Always lambed at birth site	144 (54.14)	128 (70.72)	X^2^ = 15.88, d.f. = 3, *P* < 0.001	
Individual pen after lambing (all)	191 (72.35)	58 (32.04)	X^2^ = 113.84, d.f. = 4, *P* < 0.001	
Individual pen after lambing (triplets)^c^	39 (14.77)	31 (17.13)		
Individual pen after lambing (none)	26 (9.85)	89 (49.17)		
Shelter provision (all)	170 (63.67)	92 (50.83)		
Routine colostrum supplement (all)	208 (77.90)	106 (58.56)	X^2^ = 32.37 d.f. = 4, *P* < 0.001	
Routine colostrum supplement (triplets)^c^	22 (8.23)	15 (8.28)		
Routine colostrum supplement (none)	27 (10.11)	56 (30.94)		
Inspect several times per day at lambing	262 (98.12)	124 (68.51)	X^2^ = 85.32 d.f. = 4, *P* < 0.001	
Inspect once per day or less	1 (0.37)	31 (17.13)		
Lambs assisted to suck if required	265 (99.25)	154 (85.08)	X^2^ = 39.00 d.f. = 3, *P* < 0.001	

For Y/N answers the Y responses are presented. Significant differences between countries are shown in bold.

^a^Although farmers were asked about this management practice relative to litter size most farmers did not practice differential management by litter size and thus these are not reported.

^b^Includes triplets only or twins and triplets.

^c^Triplets only or triplets and twins.

In general, farmers did not adopt a different lambing management strategy for their triplet lambs compared to singles or twins: for most attributes (Table 8) there was no clear differentiation between different litter sizes and management activities. It was apparent (Table 8A) that NZ respondents adopted a different style of management focused on low inputs and allowing ewes and lambs to develop bonds without human intervention. In contrast, UK and IRE respondents were more likely to inspect several times a day, intervene to move ewes and lambs to lambing pens, provide supplementary colostrum and assist lambs to suck. A marked difference was also seen between respondents who lambed indoors and those who lambed outdoors with indoor lambing characterized by frequent inspections, a greater use of individual lambing pens after birth, routine administration of colostrum to all lambs, assistance of lambs to suck and a lower frequency of allowing the ewe to lamb at her chosen birth site (Table 8B).

There was an impact of country on scores for Best Pregnancy Practice and Total Best Practice with UK respondents scoring the highest, and NZ respondents the lowest (Table 9, *P* < 0.001). UK and IRE respondents also scored higher for Best Triplet Lamb Practice than NZ respondents (Table 9, *P* < 0.001).

**Table 9:** Results of Best Practice Scores reported as mean and standard error, or confidence interval (CI) for respondents from UK, IRE, and NZ^*^.

	**UK**	**IRE**	**NZ**	**Significance**
Best Pregnancy Practice	4.5 ± 0.24^a^	4.2 ± 0.23^b^	3.4 ± 0.36^c^	*F*_1, 445_ = 8.77, *P* < 0.001
Best Triplet Lamb Practice	4.1 ± 0.23^a^	4.1 ± 0.20^a^	2.3 ± 0.19^b^	*F*_2, 443_ = 25.99, *P* < 0.001
**Total Best Practice**	8.8 ± 0.40^a^	8.2 ± 0.41^b^	5.8 ± 0.54^c^	*F*_2, 443_ = 21.81, *P* < 0.001

^*^Superscripts with different letters indicate significant (P ≤ 0.05) differences.

Overall, younger respondents had higher scores for Best Triplet Lamb Practice than older respondents (≤ 44 years: 3.7 ± 0.19, ≥45 years: 3.3 ± 0.19, *F*_1, 443_ = 11.56, *P* < 0.001). The same was evident for younger UK respondents with outdoor systems than older respondents (≤ 44 years: 4.3 ± 0.24, ≥45 years: 3.3 ± 0.28, *F*_1, 63_ = 7.42, *P* = 0.008). However, older UK respondents with indoor systems had higher Best Pregnancy Practice scores than younger respondents (≤ 44 years: 4.5 ± 0.24, ≥45 years: 5.2 ± 0.26, *F*_1, 101_ = 4.97, *P* = 0.028), but reported higher lamb mortality rates [≤ 44 years: 10.8 [9.7: 11.9], ≥45 years: 15.7 [14.2; 17.4], Wald = 6.94, df = 1, *P* = 0.008].

Independent of country, female respondents had higher scores for Best Triplet Lamb Practice than male respondents (Female: 3.7 ± 0.21, Male: 3.3 ± 0.19, *P* = 0.027). For IRE respondents with indoor systems, female respondents had higher Best Triplet Lamb Practice (Female: 4.9 ± 0.32, Male: 4.1 ± 0.11, *F*_1, 156_ = 7.21, *P* = 0.008) and Total Best Practice scores (Female: 9.6 ± 0.60, Male: 8.2 ± 0.19, *F*_1, 156_ = 4.63, *P* = 0.033) than male respondents. There were no gender differences (*P* > 0.05) within UK outdoor and indoor systems, or for NZ respondents.

Scores for Best Triplet Lamb Practice decreased with an increase in flock size (β = −0.0002, *P* < 0.001). Within countries, increasing flock size was positively associated with Best Pregnancy Practice score for UK respondents with indoor systems (β = 0.0007, *P* = 0.046) and IRE respondents with outdoor systems (β = 0.0034, *P* = 0.031). For NZ respondents increasing flock size was associated with a decreased score for Best Triplet Lamb Practice (β = −0.0001, *P* = 0.049).

Overall, higher scores in Total Best Practice were associated with an increased percentage of triplets born (β = 0.03935, *P* = 0.008). For IRE indoor systems, higher scores for Total Best Practice were associated with higher lambing percentage (β = 0.0473, *P* = 0.014). Higher Best Pregnancy Practice scores for UK respondents with outdoor systems were associated with increased lamb mortality (β = 0.2341, *P* = 0.008) and increased Best Triplet Lamb Practice scores were associated with an increase in lambing percentage (β = 0.0390, *P* = 0.029) and lower lamb mortality (β = 0.2461, *P* = 0.017).

### 3.4 Satisfaction with management and wanting triplet lambs

The respondents reported no overall satisfaction or dissatisfaction with their triplet management (scores of ~2.5 or the midpoint of the Likert scale). There was no effect of country in satisfaction with their triplet lamb management [mean Likert scores: UK: 2.6 [1.56; 3.66], IRE: 2.7 [1.65; 3.75], NZ: 2.7 [1.60; 3.70], *P* = 0.357]. However, all respondents marginally disagreed with the statement “Would you prefer not to have any triplet lambs?,” and this also did not differ by country [UK: 2.0 [0.83; 3.18], IRE: 1.9 [0.65; 3.00], NZ: 1.8 [0.65; 3.00], *P* = 0.339].

Older respondents were less likely to want triplets compared to younger respondents [≥45 years: 2.0 [0.95; 3.13], ≤ 44 years: 1.8 [0.67; 2.85]; *P* = 0.002]. There were no other significant effects of age, gender, or experience (data not shown). Respondents within countries (and within outdoor and indoor systems) did not differ in their satisfaction with their management practices of triplets, nor regarding if they wanted triplets or not.

## 4 Discussion

This study has demonstrated that farmers in all countries involved in the survey are interested in the management of triplet lambs and employ different practices in an attempt to overcome the higher mortality of triplet lambs. There were significant differences in management practices between countries, which was not necessarily linked to indoor or outdoor lambing system. However, it was clear that use of indoor or outdoor lambing was a feature of UK and IRE production, whereas for NZ respondents nearly all utilized only outdoor lambing, which meant that impact of lambing indoors or outdoors could not be assessed in NZ respondents. The Best Practice scores developed in this study highlighted that respondents who had more survival-specific management practices in place during pregnancy and within the first 3 days *postpartum* also had higher lamb survival rates and higher lambing percentages. Furthermore, flock size, respondent age and gender determined the level of Best Practice Scores and could help inform and tailor management strategies to improve triplet lamb survival.

### 4.1 Demographics and production statistics

As a survey study there are potentially some limitations or biases that it is important to acknowledge. The survey was only available online and thus could only have been completed by farmers who had access to the internet and were comfortable with using this method of communication. The topic of this survey was specifically related to triplet lamb management and survival. Thus, the sheep farmers that took part in the questionnaire could have been more open to new practices to improve lamb outcomes and ewe productivity and thereby not particularly representative of the population of sheep farmers in their respective countries. In fact, the average IRE and UK flock sizes in this study were approximately double the average national flock sizes of 109 or 222 breeding ewes for Ireland and the UK, respectively (40, 41) whereas NZ respondents had similar flock sizes to the average national of 2,772 (41). These numbers suggest that respondents of this study represented larger enterprises than the average of their respective countries, at least for the UK and Ireland. However, respondents with smaller flock sizes were excluded to represent sheep enterprises. Furthermore, the overall scanning percentage reported in this study was 182 with an average lambing percentage of 166. The national average lambing percentage was ~122 for the UK in 2019 (42) and 127 for NZ (43) and 137 for Ireland. As lambing percentages increase above 170 the number of single-born lambs decreases and more sets of triplets are born (44). In this study, ~9% of litters born in the 2019 lambing period were triplet lambs as reported by respondents. Although these producers were already producing more triplet lambs than the average farmer in their respective countries, use of prolific breeds e.g., Belclare, Romanov, Finn would increase mean litter size and thus the number of triplets. However, ewes with high litter size require more intensive management than is normally provided in pasture-based sheep farming which dominates global meat sheep production. The three countries UK, IRE, and NZ were chosen because they predominantly produce lamb for meat production and represent all scales of enterprise (or lambing system), from smaller more intensively managed farming enterprises to larger, lower input extensive systems and from indoor to outdoor systems. However, this meant that lambing system was highly confounded by country and distinct differences between countries were found. UK and IRE respondents represented both indoor and outdoor systems, whereas the majority of NZ respondents reported lambing outdoors and flock sizes also differed significantly between countries.

A majority of UK and IRE respondents were interested in mothering abilities of the ewe as a primary breeding goal. However, the majority of UK and IRE respondents would remove a triplet lamb from their mother and adopt it to another ewe or for artificial rearing, indicating higher labor input in their systems. Reasons for lamb removal (e.g., maternal milk supply) were not explored in this study, and mothering ability could include both maternal care and milking ability, but if three lambs are to be reared by a ewe then her ability to do this is an important consideration. In comparison to the UK and IRE, NZ respondents had number of lambs reared as a main breeding goal and triplet lambs would primarily stay with the ewes. The temperate climate in NZ enables year round pasture availability for outdoor lambing, and easy-care lambing was developed in NZ to improve lamb survival (24). Furthermore, large flock sizes and challenging extensive hill country landscapes limit intensive management practices. Thus, breeding goals of NZ farmers have for decades focused on breeding for mothering abilities and lamb vigor, which may explain why mothering ability was not ranked highest as it already is embedded in their breeding goals. An interesting difference emerged between countries in terms of which lamb would be removed from the litter to reduce the litter to two lambs. NZ respondents were more likely than UK or IRE respondents to select a medium-sized lamb or the weakest lamb. This latter result might partially be explained by the greater likelihood of triplet lambs staying with the ewe and thus only those lambs that seemed unlikely to survive without additional support would be removed in NZ. The fact that UK and IRE respondents would almost always take a triplet lamb from its mother, whereas NZ respondents would be more likely to leave all triplets with the dam could also explain the tendency for differences by country in lamb mortality, defined as lambs lost between scanning and 12 weeks of age in this study. In a study evaluating ewe and lamb performance of triplet-bearing ewes where all triplets were kept on the ewe or the litter was reduced to two lambs, lamb mortality was also significantly higher for ewes who had all three lambs at foot (9). Thus, the more intensive approach of triplet lamb management by UK and IRE respondents in this study may increase survival rates of triplet lambs, which may explain the differences in lambs lost between scanning and 12 weeks of age compared to NZ respondents. Notably, the obtained data of lambs lost between scanning and 12 weeks of age was not only for triplets but for all lambs in the flock and also included lambs lost before birth. A consideration in the interpretation of the questionnaire was that the data collected in the study were reported data, meaning that farmers reported scanning and lambing percentages as well as number of triplets born in their last lambing period, and these could not be corroborated. Adam et al. highlighted especially the stigma around recording losses to avoid negative emotions and fear of judgment from peers (45), which may also have been an issue in this study, even though this survey was confidential.

Previous studies similar to this one identified higher lamb mortality rates in outdoor systems compared to indoor systems (46, 47), but mortality can vary significantly among flocks (48). A New Zealand study reported that mortality rates of 10 farms varied from 1.4 to 43.5% and also varied between years (49). A recent study reported mortality rates of triplet lambs to be 41% in Australia (50). Thus, this study indicates significantly lower triplet lamb mortality in the three countries investigated compared to Lockwood et al. (50), although management practices and conditions do vary in this study.

### 4.2 Best practice

The aim of developing the Best Practice scores was to identify influencing factors of triplet lamb management which may improve lamb survival. Higher scores for Best Pregnancy Practice would potentially optimize fetal growth and development as well as ewe health and body condition, whereas the Best Triplet Lamb Practice score focused on management practices during parturition and in the early postnatal period to support lamb survival. This included supervision to provide birth assistance or assisting lambs to the udder if needed, as previous research has suggested that low vigor in triplet lambs is a contributor to poor survival (51). The most critical time period of lamb survival is within the first 72 h after birth with ~75% of all mortalities occurring in this time frame (52). The risk of dystocia, hypothermia, starvation, mismothering, and infectious disease due to inadequate colostrum intake are higher for triplet lambs than for twin- and single-born lambs in this period (11). Therefore, in this study, the focus was on the first 3 days after birth, taking both indoor and outdoor lambing practices into account by giving respondents the option to assist ewes at birth, choose use of a lambing pen or shelter, and assisting lambs to the udder or tube feeding if needed. The Best Practice scores were developed as additive models, whereby every practice performed by the respondent was weighted equally with others. Other approaches could have been chosen such as weighing certain practices higher than others. However, to avoid bias toward or against certain practices for indoor and outdoor systems, the practice of either providing a pen or shelter to new-born triplet lambs was considered to be equivalent and relevant for respondents from either indoor or outdoor lambing systems. It was recognized that not all farmers could fulfill all criteria. The respondents were able to identify their management practices to different scenarios regarding the provision of a pen, choosing to move the ewe prior to lambing or after lambing. However, this question could have been misunderstood. The birth site is important for the development of the ewe-lamb bond and hours before giving birth, the ewe will have chosen a birth site which she will not leave (12). Choosing to include the category that lambs and ewes are moved to a pen after birth was based on potential stocking rates in the lambing pen for indoor systems, the risk of mismothering and caretaker hygiene measures. However, it should be acknowledged that ewe behaviors were not taken into account and that farmers could pen their ewes *in situ* if needed. A timeframe for when this would be undertaken could have been included. In a survey study by Mahon et al. (53) it was highlighted that “Best Practice” can vary from farm to farm, meaning that some practices may not be appropriate or possible. This was also reflected in this study; indoor and outdoor lambing sheep farms differ, and individual sheep farms differ from one another even with a similar lambing system.

Labor input has been described as the biggest contrast between intensive and extensive systems (24). However there can be additional factors related to indoor and outdoor lambing, such as flock size, terrain, space and social norms. The results of the present study underline that outdoor lambing systems may not be characterized by lower inputs *per se* and can be highly managed systems. In the present study 39% of UK respondents reported to lamb outdoors; and UK respondents overall had the highest scores for Best Pregnancy Practice and Best Triplet Lamb Practice compared to IRE and NZ respondents. Furthermore, UK outdoor systems, which had higher Best Triplet Lamb Practice scores, had lower lamb mortality rates and increased lambing percentages. However, there was an anomalous result where UK farmers lambing outdoors with high Best Pregnancy Practice scores did not have higher lamb survival. This may suggest that our Best Pregnancy Practice may not have captured all the elements of pregnancy management outdoor that can achieve higher lamb survival (such as methods to manage ewe nutrition outdoors or health management). In relation to the present study, a previous survey of UK farmer practices using either indoor or outdoor lambing systems, found that most farmers suggested that more supervision at lambing time would have an impact on the welfare of their animals (54). Thus, UK farmers, independent of lambing system, consider that labor use and supervision are important aspects of lambing management. This study also emphasized that UK extensive systems were not characterized by lower management compared to indoor systems (54), which is supported by the present study where farmers with outdoor systems also had high Best Practice scores. This finding highlights firstly, the capability to perform lambing assistance, assisting lambs to the udder and administering colostrum in both indoor and outdoor systems (22), and secondly, highlights the perceived need for a greater labor input, at least in some systems. Previous studies have questioned whether shepherding would be beneficial in outdoor systems as it could negatively affect the ewes' natural behaviors to select a birth site and the development of the ewe-lamb bond (55). The results for the Best Triplet Lamb Practice scores from NZ respondents in the present study could indicate that, for outdoor systems, human interventions are intentionally kept to a minimum to avoid disturbance. In these systems, other aspects of risk management, such as ensuring optimal ewe nutrition, a suitable lambing environment and adopting good health care practices, can reduce the need for high levels of human intervention.

Flock size had a significant positive effect on scores for Best Pregnancy Practice for UK indoor and IRE outdoor systems. This may be because, as more ewes in the flock decreases the labor input/time per ewe (56, 57), farmers invest in other approaches to facilitate ewe management, such as improved farm infrastructure or adoption of digital and other management tools. Similar results were found in survey studies conducted on sheep farmers from IRE and NZ where farmers with larger sheep flocks were more likely to have additional infrastructure such as handling facilities to reduce labor input or were more likely to adopt new farm management tools (58, 59). However, increased flock size was also associated with increasing lamb mortality and decreasing Best Triplet Lamb Practice in the present study. Increasing numbers of ewes lambing in indoor systems have previously been associated with a higher risk of lamb mortality (22). Larger flock sizes have been linked to increased infectious disease and trauma in indoor lambing systems associated with increased stocking rates, lack of supervision of lambing ewes and lack of time to keep hygiene measures in place (12). Similar to indoor lambing systems, many outdoor systems with greater flock size could struggle to provide sufficient support to reduce starvation-exposure, dystocia and mismothering, if labor is not also increased (16, 60). With more ewes giving birth to larger litters, it may not be possible to uphold the traditional system of no intervention to reduce lamb mortality, and shepherding could contribute significantly to improving animal welfare (24, 61).

The questionnaire identified that younger respondents scored higher for Best Triplet Lamb Practice than older respondents. The results suggest that younger respondents may be more open to engaging with and acting on advice and information. In a NZ survey study investigating factors affecting the use of digital farm management tools by sheep farmers, farmer age was also a significant factor (59). Younger respondents were more likely to make use of management tools than older respondents (62). This may be because younger respondents were more familiar and confident with digital methods to access knowledge, may have access to methods to share information more readily and their knowledge of innovation in sheep farming may therefore be up to date compared to older respondents. They may also be more willing to explore new methods than older respondents who can be less likely to adopt new practices. Older farmers are reported to be less inclined to adopt to new strategies (37) which may be due to a reduced planning horizon, fewer incentives to change, and less exposure to new knowledge (62). The effect of education has also been identified to be a significant factor influencing the use and adoption of management tools (62, 63). However, in this study, 79% of respondents had educational backgrounds that included some post school education (further or higher education), suggesting that respondents of this study were highly educated. This may suggest that our respondents had the capability to adapt to management practices that improve lamb survival but requires further investigation as whether the respondents had education specifically in farming or agriculture was not known. Furthermore, the higher Best Triplet Lamb Practice scores achieved by younger respondents could explain the lower lamb mortality rates reported by younger respondents than older respondents. Additionally, older farmers may be less likely to be influenced by social expectations and more focused on financial performance than younger farmers, independent of educational status, in relation to values and decisions related to environmental impact of farming (64). Thus, the financial impact of sheep farming may also have played an important role for older sheep farmers who took part in the survey. In general, the average age of sheep farmers is high, being above 50 (58, 65, 66). However, in this study, 49% of respondents were 44 years and younger, and 51% were 45 years and older. This may be because younger respondents were more actively seeking to increase their flock prolificacy than older farmers, and therefore the response group was biased toward a younger cohort who were interested in triplet lamb management. However, this may also have been affected by the dissemination of the questionnaire online and that invitation to the survey was through both social and mainstream media.

Overall, female respondents in the current study scored higher for Best Triplet Lamb Practice than male respondents, and within IRE indoor systems female respondents had higher Best Practice scores than male respondents. Interestingly, female respondents also reported slightly lower lambing percentages and fewer triplets as a proportion of their flocks compared to males. Females have been found to be more concerned about the welfare of the individual animal, and welfare in general than males (67–69). In an Australian survey study investigating welfare perceptions of sheep farming, women were more likely to rate welfare issues to be a cause of compromise than men (70). Furthermore, previous research indicates that women place greater significance on painful procedures in animals and are more likely to rate this as compromised welfare compared to men (71). Thus, women in this study may be more aware of the welfare benefits of providing additional care to triplets and may be more motivated to provide care to the individual triplet lamb.

The emotional impact of mortality on farmer wellbeing was not investigated in this survey. Adam et al. (45) highlighted the financial, social, and emotional impact of lamb mortality where sheep farmers reported frustration, guilt, and fear of judgment from other farmers in response to lamb losses. The aspect of stress in farmers has previously been highlighted (72–74). In a NZ survey study, it was highlighted that most stress came from increasing workload at peak times and bad weather (73). In this study, although individual views of their satisfaction with their management of triplet lambs varied, on average farmers were neither satisfied nor dissatisfied with their management. This suggests that there may be scope for improvements in management to achieve greater satisfaction, which can contribute to farmer wellbeing. In addition, wanting higher lambing percentages may also depend on the financial and emotional stability of each farmer, as it requires greater resource input (e.g., labor, infrastructure and artificial rearing costs) and may result in “burn-out” and frustration, guilt, and financial worries. Therefore, an important question of this survey was whether respondents wanted triplet-lambs on their farm. Increasing the number of lambs born per ewe has the potential to increase farm profitability (75) which could be an incentive to increase lambing percentages, but the additional inputs and higher mortality of triplet lambs may counter these benefits. This survey showed that younger respondents were more likely to want triplets than older respondents, and there was no difference between the countries surveyed. Interestingly, as the younger respondents also had higher Best Triplet Lamb Practice scores than older, younger respondents of this survey may also be aware of the greater workload it requires or consider that they had the physical and mental capacities to achieve higher triplet lamb survival.

## 5 Conclusion

This study identified significant differences in farming systems between countries and within countries, particularly with respect to the management of larger litter sizes. Country of origin, age, and gender of respondents as well as flock size were distinct factors that affected management practices of triplet-bearing ewes and lambs. In particular, there was a marked country effect determining whether triplet lambs were all left with the ewe to rear, or whether one lamb was removed to rear artificially or to adopt onto another ewe. This may be influenced by farming “cultures” in different countries between low input systems that require animals to be self-sufficient, compared to more interventionist approaches. These “cultures” may in turn be driven by structural issues such as terrain, land mass, and pasture availability in each country. The respondents to this survey were younger, more highly educated and managed flocks of sheep that may have been more productive (for example higher scanning percentages) than the national average for each country. As it was an online survey this may have limited responses to farmers who were comfortable with this technology. This may have led to some bias in the results in terms of extrapolating to all farmers in each country. However, the purpose of the study was to determine what the most progressive farmers, who had experience of managing triplet lambs, were doing in terms of their management. This may provide an indicator of the responses of “industry leaders” in this area and so is valuable to understand potential future trends. Thus, the study did provide insights into the activities and goals of this group of farmers, who were focused on high levels of productivity. The results of this study could inform ways of tailoring knowledge transfer to farmers country of origin, age, gender, and flock demographics to improve triplet lamb survival.

## Data availability statement

The data presented in the study are deposited in the SRUC repository, https://doi.org/10.58073/SRUC.25118777.v2.

## Ethics statement

The questionnaire was approved by the Royal (Dick) School of Veterinary Studies Human Ethical Review Committee (approval number: HERC_415_19) and AgResearch Human Research and Ethical Conduct (approval number: 14/2019).

## Author contributions

CE: Conceptualization, Data curation, Formal analysis, Investigation, Methodology, Project administration, Validation, Visualization, Writing – original draft, Writing – review & editing. TC: Conceptualization, Data curation, Formal analysis, Methodology, Supervision, Writing – review & editing. NS: Conceptualization, Data curation, Methodology, Supervision, Writing – review & editing. SM: Conceptualization, Data curation, Funding acquisition, Methodology, Supervision, Writing – review & editing. TK: Conceptualization, Data curation, Formal analysis, Supervision, Writing – review & editing. CD: Conceptualization, Data curation, Formal analysis, Funding acquisition, Methodology, Resources, Supervision, Writing – review & editing.

## Footnotes

^1^ https://cordis.europa.eu/project/id/727895

^2^ https://www.jisc.ac.uk/
