# Supplementary material for: Improving triplet lamb survival: management practices used by commercial farmers
Source: Front Vet Sci. 2024 Jul 30;11:1394484. doi: 10.3389/fvets.2024.1394484 (PMC11319296; doi:10.3389/fvets.2024.1394484)
Supplement: Supplementary file 1 [file Data_Sheet_1.PDF]

Triplet lamb survey

Page 1: Consent

Hello, welcome and thank you for showing interest in this survey on triplet-lamb management practices in New Zealand, the United Kingdom and Ireland.

My name is Cathrine Erichsen and this survey is part of my PhD research project investigating management and survival of triplet lambs. In this survey we are interested in your opinion about triplet lambs, what management might be needed to keep lambs alive, and whether triplet lambs are part of your goals for your farming practice. This project is funded by the AgResearch Strategic Science Investment Fund (SSIF) with stipend support from the AgResearch SSIF International Linkage Fund and SRUC. This study is a collaboration between Scotland's Rural College (SRUC) (United Kingdom), AgResearch (New Zealand), the University of Edinburgh (United Kingdom) and Teagasc (Ireland).

The survey will take approximately 15 minutes to complete.

Your participation in this survey is completely voluntarily and you may withdraw at any time by closing this browser window. The information given in the survey is completely anonymous, and the data collected in this survey will not be able to identify you as an individual, we are interested in understanding the general practice of triplet-lamb management.

As the survey is anonymous it will not be possible to your answers once you have

submitted. However, all data will be destroyed 10 years after the end of the evaluation of the project. Further information can be obtained from Cathrine Erichsen DVM, PhD student at SRUC, AgResearch and University of Edinburgh.

cathrine.erichsen@sruc.ac.uk or cathrine.erichsen@agresearch.co.nz

My research is supervised by Prof Cathy Dwyer (SRUC), Dr Sue McCoard (AgResearch), Prof Neil Sargison (Royal (Dick) School of Veterinary Studies) and Dr Tamsin Coombs (SRUC).

1. I understand the purpose and nature of this study and I am participating voluntarily. I understand that I can withdraw from the study at any time, without any consequences. *Required*

- Yes

Page 2: About you

2. Where do you live? □ *Required*

- ◯ New Zealand
- ◯ United Kingdom
- ◯ Ireland

2.a. Please indicate the first section of your postcode if you live in the UK (eg EH)

2.b. Please tell where you live in New Zealand

2.c. In which Irish county do you live?

3. I describe myself as □ *Required*

- ◯ Female
- ◯ Male
- ◯ In another way
- ◯ Prefer not to say

4. What is your age? □ *Required*

- under 25 years old
- 25 - 34 years old
- 35 - 44 years old
- 45 - 54 years old
- 55 - 64 years old
- 65 years or older

5. What is your highest level of education completed? □ *Required*

- High School
- Further education
- University level
- Other

5.a. If you selected Other, please specify:

6. For how many years have you worked with sheep as your primary occupation? □ *Required*

- Less than 5 years
- 5-10 years
- 10-20 years
- More than 20 years

7. Which answer describes you best? *Required*

- ◯ Farm owner and manager
- ◯ Farm owner
- ◯ Farm manager
- ◯ Farm tenant
- ◯ Farm staff
- ◉ Other

7.a. If you selected Other, please specify:

8. Are you one of the primary decision makers for management on the farm? *Required*

- ◯ Yes
- ◯ No

Page 3: About your farm

9. How do you describe your farm? *Required*

- ○ Sheep farm
- ○ Dairy sheep farm
- ○ Combined sheep and beef farm
- ○ Combined sheep and crop farm
- ○ Combined sheep and deer farm
- ○ Combined sheep, beef and crop farm
- ○ Combined sheep, beef and deer farm
- ● Other

9.a. If you selected Other, please specify:

10. Is your flock? *Required*

- ○ Commercial flock
- ○ Stud/pedigree flock
- ○ Commercial and stud/pedigree flock

11. Is your farm (tick all that apply) *Required*

- ☐ Unfenced, unimproved pasture all year round (never housed)
- ☐ Unfenced, unimproved pasture part of the year
- ☐ Fenced, pasture all year round (never housed)
- ☐ Fenced pasture part of the year
- ☐ Housing for part of the year
- ☐ Housing all year round

Page 4: About your flock

For the purposes of the rest of this survey please think about the last complete lambing season (joining/mating until weaning). If you have more than one farming system on your farm please consider the system where you have most triplet lambs.

12. How many sheep (total number of female sheep)) did you put to the ram? □ *Required*

12.a. Of the total, how many hoggets/ewe lambs did produce their first litter as 1 year olds? □ *Required*

12.b. Of the total, how many gimmers/two-tooths (2 years of age) did you lamb? □ *Required*

13. What breed or type of ewes are in your flock (eg Romney, Commercial Cross Breed, Composite)? □ *Required*

14. What breed or type of rams are in your flock (eg. Romney, Commercial Cross Breed, Composite)? *Required*

15. In which month of the year were most lambs born in your most recent lambing season (January, February, March, April, May, June, July, August, September, October, November, December)?

Required

16. Do you scan for pregnancy? *Required*

- Yes
- No

16.a. If you answered yes in the last question, do you pregnancy scan:

- Barren/in lamb
- Single/multiple
- Litter size
- Litter size and fetal age

16.b. What was the scanning percentage at your last lambing season?
(scanning percentage is defined as number of lambs scanned in the ewes per number of ewes put to the ram x 100)

16.c. What opinion do you have about your scanning percentage?

- It's too high
- It's too low
- It's optimal

16.d. What is your opinion on the statement below?

	I strongly disagree	I disagree	Neither agree nor disagree	I agree	I strongly agree
There is an economic advantage in high scanning percentages					

17. How many sets of multiples (triplets and quads) were born in your last lambing? (enter a number, range, or 'don't know') □ *Required*

18. What was the lambing percentage at your last lambing? (lambing percentage is defined as number of lambs per number of ewes (or present at 12 weeks) put to the ram x 100) □ *Required*

Page 5: Management of pregnant ewes

For the purposes of the rest of this survey please think about the last complete lambing season (joining/mating until weaning). If you have more than one farming system on your farm please consider the system where you have most triplet lambs.

19. Please choose your three most important breeding criteria *Required*

Please select no more than 3 answer(s).

- ☐ Geographic/climate suitability
- ☐ Resilience to disease
- ☐ Number of lambs scanned
- ☐ Number of lambs born
- ☐ Number of lambs reared
- ☐ Birth ease
- ☐ Mothering abilities
- ☐ Milk yield
- ☐ Lamb vigour
- ☐ Muscularity
- ☐ Growth rate
- ☐ Low cost input
- ☐ Other

19.a. If you selected Other, please specify:

20. Do you body condition score? *Required*

- Visually
- By manual palpation
- I don’t body condition score
- Other

20.a. If you selected Other, please specify:

21. How do you manage your ewes during pregnancy? (tick all that apply) *Required*

- Ewes are fed according to litter size
- Ewes are managed according to body condition score (BCS)
- Triplet-bearing ewes are kept in separate groups
- Ewes are frequently inspected
- Ewes are supplemented in the last 4-6 weeks of pregnancy
- Ewes are shorn prior to lambing
- Other

21.a. If you selected Other, please specify:

Page 6: Comparisons of lamb management

For the purposes of the rest of this survey please think about the last complete lambing season (joining/mating until weaning). If you have more than one farming system on your farm please consider the system where you have most triplet lambs.

22. How do you manage your single, twin and triplet-bearing ewes and lambs around lambing? (tick all that apply) □ *Required*

	Single	Twins	Triplets	No
Ewes are moved daily prior to lambing				
Ewes are set-stocked at lambing				
Pregnant ewes are moved and lambing ewes are left				
Ewes are left alone for lambing				
Ewes are allowed to lamb at the birth site				
Ewes are moved to a lambing pen prior to lambing (not at the birth site)				
Ewes and lambs are moved to a pen after birth				
Ewes and lambs are provided with shelter				
All lambs are always supplemented with colostrum				

Lambs that have not sucked from the ewe are supplemented with colostrum				
Ewes and lambs are kept in individual pens after birth				
Lambs are given antibiotics				

Page 7: Lambing management

For the purposes of the rest of this survey please think about the last complete lambing season (joining/mating until weaning). If you have more than one farming system on your farm please consider the system where you have most triplet lambs.

23. How often do you inspect your ewes around the time of lambing? *Required*

- Hourly checks
- Several times a day
- Twice a day
- Once a day
- Less than once a day

23.a. If you answered 'less than once a day' please specify how often you inspect around the time of lambing.

24. What is your first choice for assisting lambs that haven’t sucked? □ *Required*

- ○ By holding the lamb to the teat
- ○ By giving colostrum by stomach tube
- ○ By giving colostrum by bottle
- ○ I don’t assist

25. What is your preferred supplement? (tick all that apply) □ *Required*

- □ Ewe colostrum
- □ Cow’s colostrum
- □ Artificial colostrum
- □ I don’t supplement
- □ Other

25.a. If you selected Other, please specify:

Page 8: Triplet-lamb management

For the purposes of the rest of this survey please think about the last complete lambing season (joining/mating until weaning). If you have more than one farming system on your farm please consider the system where you have most triplet lambs.

26. What is your general management practice for **triplet-born** lambs? □ *Required*

- I keep all triplets on the ewe
- I artificially rear one triplet
- I adopt one triplet off
- I sell one triplet
- I dispose of one triplet
- Other

26.a. If you selected Other, please specify:

27. If you take a **triplet-lamb** off the ewe which lamb do you generally choose? □

- The smallest/lightest lamb
- The medium sized/weight lamb
- The biggest/heaviest lamb
- The weakest lamb regardless of size/weight
- The most vigorous lamb regardless of size/weight
- A male
- A female
- Other

27.a. If you selected Other, please specify:

28. To what extent are you satisfied with your triplet management? □ *Required*

- Very dissatisfied
- Dissatisfied
- Neither satisfied nor dissatisfied
- Satisfied
- Very satisfied

29. Feel free to add information about triplet-lamb management which has not been covered in the questions above. *Optional*

Page 9: Opinions on costs

For the purposes of the rest of this survey please think about the last complete lambing season (joining/mating until weaning). If you have more than one farming system on your farm please consider the system where you have most triplet lambs.

30. What is your opinion on the statements below? *Required*

	I strongly disagree	I disagree	Neither agree nor disagree	I agree	I strongly agree
The cost for pregnancy scanning for better triplet-lamb management is justifiable					
The highest expense of triplet-lamb management is feeding the pregnant ewe					
Working hours at lambing related to triplet-lamb management are too					

high					
Staff costs at lambing for triplet-lamb management are justifiable					
Artificially rearing triplet lambs is too expensive					

The cost for supplementing triplet ewes and their lambs is justifiable					
The biggest cost in triplet-lamb management are lamb losses					
The biggest cost in triplet-lamb management are ewe losses					
The overall cost of triplet-lamb management is too high for me					

31. Would you prefer not to have any triplet-lambs born on your farm? *Required*

- I strongly disagree
- I disagree
- Neither agree nor disagree
- I agree
- I strongly agree

31.a. Why did you answer ‘highly disagree’, or ‘disagree’ in the previous statement?

Page 10: Opinions on triplet-lamb management

32. What is your opinion on the statements below? □ *Required*

	I strongly disagree	I disagree	Neither agree nor disagree	I agree	I strongly agree
Pregnant ewes should be fed according to number of fetuses carried					
Body condition scoring the ewes during pregnancy is essential					
Intensive shepherding is required to keep triplets alive					
Handling the ewes and lambs at lambing should be minimised					
Triplet-bearing ewes should be lambed indoors					
Shelter should be provided					

Good weather conditions are needed					

Page 11: Opinions on triplet-lamb and ewe welfare and health

33. What is your opinion on the statements below? □ *Required*

	I strongly disagree	I disagree	Neither agree nor disagree	I agree	I strongly agree
Triplet lambs are as healthy as twins					
Triplet lambs are at higher risk of suffering from pain, fear or distress than twin-born lambs					
Triplet lambs are as well-nourished as twin-born lambs					
Triplet lambs are more likely to experience hunger or thirst than twin-born lambs					
Triplet lambs are more likely to experience more discomfort such as cold than twin-born lambs					

Triplet lambs express the same behaviours like twins					

34. What is your opinion on the statements below? □ *Required*

	I strongly disagree	I disagree	Neither agree nor disagree	I agree	I strongly agree
Carrying and nursing triplet lambs will decrease the longevity of the ewe					
Ewes which carry triplets are less likely to lamb the following year					
Ewes which have triplets will have more living lambs over their lifetime than ewes which do not					

Page 12: Final page

Thank you for your participation, it is much appreciated.

The results from this survey will be published and aim to contribute to triplet-lamb survival research. If you are interested in the results from this survey, you are welcome to email me on

cathrine.erichsen@sruc.ac.uk or cathrine.erichsen@agresearch.co.nz

Yours, sincerely

Cathrine Erichsen, **SRUC, AgResearch, University of Edinburgh**
