# Supplementary material for: Improving triplet lamb survival: management practices used by commercial farmers
Source: Front Vet Sci. 2024 Jul 30;11:1394484. doi: 10.3389/fvets.2024.1394484 (PMC11319296; doi:10.3389/fvets.2024.1394484)
Supplement: Supplementary file 2 [file Table_1.DOCX]

Table 1: Table listing the grouping decision made for demographic and management responses to balance the data.

| Category | Original category points | New developed category points |
| --- | --- | --- |
| Gender | Female  Male  In another way  Prefer not to say | Female  Male  * (reclassified as missing and not reported) |
| Age of respondent | 25 years and younger  26-34 years  35-44 years  45-54 years  55-64 years  65 years and older | ≤44 years  ≥45 years |
| Experience | Less than 5 years  5-10 years  11-20 years  More than 20 years | ≤10 years  ≥11 years |
| Farm type | 1. Sheep farm 2. Dairy sheep farm 3. Combined sheep and beef farm 4. Combined sheep and crop farm 5. Combined sheep and deer farm 6. Combined sheep, beef and crop farm 7. Combined sheep, beef, and deer farm 8. Other | Sheep (1,2)  Sheep and beef (3)  Sheep and other (4,5,6,7,8) |
| Body condition score | Visually  By manual palpation  I don’t body condition score  Other | Visually  By manual palpation  I don’t body condition score |
| Triplet management | I keep all triplets on the ewe  I artificially rear one triplet  I adopt one triplet off  I sell one triplet  I dispose of one triplet  Other | I keep all triplets on the ewe  I artificially rear one triplet  I adopt one triplet off  Other |
| Are singles, twins or triplet ewes and lambs moved to a pen after birth | Tick all that apply | Singles and/or twins  Twins and triplets  Triplets only  None |
| Are singles, twins or triplet ewes and lambs provided with shelter | Tick all that apply | Singles and/or twins  Twins and triplets  Triplets only  None |
| Are singles, twins or triplet lambs assisted when they have not sucked from the ewe | Tick all that apply | Singles and/or twins  Twins and triplets  Triplets only  None |
| The preferred supplement | Ewe colostrum  Cow colostrum  Artificial colostrum  No colostrum  Other colostrum | Only ewe colostrum  Ewe colostrum and something else  Something else |
| To what extent are you satisfied with your triplet management (Likert scale) | Very dissatisfied  Dissatisfied  Neither satisfied nor dissatisfied  Satisfied  Very satisfied | Dissatisfied  Neither satisfied nor dissatisfied  Satisfied |
| Would you prefer not to have any triplet lambs born on your farm (Likert scale) | I strongly disagree  I disagree  Neither agree nor disagree  I agree  I strongly agree | I disagree  Neither agree nor disagree  I agree |
